# Association of hyperglycemia and molecular subclass on survival in IDH-wildtype glioblastoma

**DOI:** 10.1093/noajnl/vdac163

**Published:** 2022-10-11

**Authors:** Elisa K Liu, Varshini Vasudevaraja, Vladislav O Sviderskiy, Yang Feng, Ivy Tran, Jonathan Serrano, Christine Cordova, Sylvia C Kurz, John G Golfinos, Erik P Sulman, Daniel A Orringer, Dimitris Placantonakis, Richard Possemato, Matija Snuderl

**Affiliations:** NYU Grossman School of Medicine, New York, NY, USA; NYU Grossman School of Medicine, New York, NY, USA; Department of Pathology, NYU Langone Health, New York, NY, USA; NYU Grossman School of Medicine, New York, NY, USA; Department of Pathology, NYU Langone Health, New York, NY, USA; Department of Biostatistics, NYU School of Global Public Health, New York, NY, USA; NYU Grossman School of Medicine, New York, NY, USA; Department of Pathology, NYU Langone Health, New York, NY, USA; NYU Grossman School of Medicine, New York, NY, USA; Department of Pathology, NYU Langone Health, New York, NY, USA; NYU Grossman School of Medicine, New York, NY, USA; Department of Neurology, NYU Langone Health, New York, NY, USA; The Laura and Isaac Perlmutter Cancer Center at NYU Langone Health, New York, NY, USA; NYU Grossman School of Medicine, New York, NY, USA; Department of Neurology, NYU Langone Health, New York, NY, USA; The Laura and Isaac Perlmutter Cancer Center at NYU Langone Health, New York, NY, USA; NYU Grossman School of Medicine, New York, NY, USA; Department of Neurosurgery, NYU Langone Health, New York, NY, USA; The Laura and Isaac Perlmutter Cancer Center at NYU Langone Health, New York, NY, USA; NYU Grossman School of Medicine, New York, NY, USA; Department of Radiation Oncology, New York, NY, USA; The Laura and Isaac Perlmutter Cancer Center at NYU Langone Health, New York, NY, USA; NYU Grossman School of Medicine, New York, NY, USA; Department of Neurosurgery, NYU Langone Health, New York, NY, USA; The Laura and Isaac Perlmutter Cancer Center at NYU Langone Health, New York, NY, USA; NYU Grossman School of Medicine, New York, NY, USA; Department of Neurosurgery, NYU Langone Health, New York, NY, USA; The Laura and Isaac Perlmutter Cancer Center at NYU Langone Health, New York, NY, USA; NYU Grossman School of Medicine, New York, NY, USA; Department of Pathology, NYU Langone Health, New York, NY, USA; The Laura and Isaac Perlmutter Cancer Center at NYU Langone Health, New York, NY, USA; NYU Grossman School of Medicine, New York, NY, USA; Department of Pathology, NYU Langone Health, New York, NY, USA; The Laura and Isaac Perlmutter Cancer Center at NYU Langone Health, New York, NY, USA

**Keywords:** glioblastoma, glucose, hyperglycemia, methylation, subclass

## Abstract

**Background:**

Hyperglycemia has been associated with worse survival in glioblastoma. Attempts to lower glucose yielded mixed responses which could be due to molecularly distinct GBM subclasses.

**Methods:**

Clinical, laboratory, and molecular data on 89 IDH-wt GBMs profiled by clinical next-generation sequencing and treated with Stupp protocol were reviewed. IDH-wt GBMs were sub-classified into RTK I (Proneural), RTK II (Classical) and Mesenchymal subtypes using whole-genome DNA methylation. Average glucose was calculated by time-weighting glucose measurements between diagnosis and last follow-up.

**Results:**

Patients were stratified into three groups using average glucose: tertile one (<100 mg/dL), tertile two (100–115 mg/dL), and tertile three (>115 mg/dL). Comparison across glucose tertiles revealed no differences in performance status (KPS), dexamethasone dose, MGMT methylation, or methylation subclass. Overall survival (OS) was not affected by methylation subclass (*P* = .9) but decreased with higher glucose (*P* = .015). Higher glucose tertiles were associated with poorer OS among RTK I (*P* = .08) and mesenchymal tumors (*P* = .05), but not RTK II (*P* = .99). After controlling for age, KPS, dexamethasone, and MGMT status, glucose remained significantly associated with OS (aHR = 5.2, *P* = .02). Methylation clustering did not identify unique signatures associated with high or low glucose levels. Metabolomic analysis of 23 tumors showed minimal variation across metabolites without differences between molecular subclasses.

**Conclusion:**

Higher average glucose values were associated with poorer OS in RTKI and Mesenchymal IDH-wt GBM, but not RTKII. There were no discernible epigenetic or metabolomic differences between tumors in different glucose environments, suggesting a potential survival benefit to lowering systemic glucose in selected molecular subtypes.

**Key Points**

- High glucose is associated with shorter OS in GBM.
- The effect of hyperglycemia is molecular subtype dependent.
- Future trials targeting glucose levels in GBM should incorporate molecular sub-classification.

**Importance of the Study**

Glioblastoma is a heterogenous disease entity with poor outcomes. While analysis of the methylation and transcriptomic landscape has revealed distinct tumor subclasses, the prognostic value of the subclasses remains unknown. Prior studies have investigated the role of glucose and glucose-lowering agents with mixed results. We demonstrate the role of hyperglycemia on glioblastoma survival in a subclass-specific manner, with RTK I and Mesenchymal tumors demonstrating worse outcomes with higher glucose. Future studies examining the role of glucose-lowering agents may incorporate methylation profiling for patient stratification.

Glioblastoma (GBM) is the most common malignant primary brain tumor and, despite aggressive multimodal therapies, has poor prognosis with an average survival of 16 months.^1^ Despite success in early-phase investigations, standard of care has remained the same since adoption of the Stupp protocol in 2005, with minimal impact of precision medicine advances on patient outcomes.^2^ While two new therapies have been FDA-approved for recurrent GBM,^3,4^ few factors have been associated with improved patient outcomes.

While hyperglycemia has been associated with increased morbidity and mortality in a variety of cancers, the role of hyperglycemia and utility of lowering serum glucose in GBM patients is controversial. Most studies suggest that hyperglycemia is an independent poor prognostic marker for overall survival (OS).^5–7^ However, investigations on glucose-lowering agents, notably the anti-diabetic drug metformin, have yielded mixed results.^8,9^ Ketogenic diets, similarly motivated by the seemingly detrimental effect of hyperglycemia, have also yet to demonstrate convincing survival benefit, possibly related to the minimal effect of low-carbohydrate diets on serum glucose.^10,11^ Despite a multitude of literature describing the poor prognosis carried by hyperglycemia, there lacks a consensus on how oncologists may leverage this finding to improve patient outcomes.

Recent advances and application of genome-wide profiling have highlighted molecular heterogeneity of GBM. Transcriptomic profiling on The Cancer Genome Atlas (TCGA) datasets revealed three main molecular GBM subgroups (Classical, Proneural, Mesenchymal) with distinct patterns of genomic aberrations and tumor evolution.^12^ Epigenomic profiling offers a complementary way of understanding tumor heterogeneity and classification using smaller samples of variable quality, thus proving to be a widely-utilized classification tool for CNS cancers.^13^ In GBM, methylation profiling classifies GBM into six distinct subgroups which correlate to TCGA subgroups including RTK II “Classic”, RTK I “Proneural”, and Mesenchymal subclasses. However, despite the improved understanding of tumor biology, the clinical relevance of these classifications remain debated.^14,15^

In our study, we sought to investigate the role of serum glucose on isocitrate dehydrogenase (IDH)-wildtype glioblastoma survival in a methylation subclass-specific manner. We hypothesized that glucose has a different role in different tumor subclasses which may have confounded prior investigations on the effect of lowering glucose. We further sought to characterize phenotypic differences of tumor subtypes associated with varying glucose environments by comparing methylation landscape and intratumoral metabolite levels.

## Methods

### Patient Cohort

Newly diagnosed patients operated and followed for GBM at NYU Langone Health between 2014 and 2020 were included in the study. As part of their clinical care, all tumors underwent clinically validated next-generation sequencing (NGS), and clinically validated whole-genome DNA methylation profiling as described previously^13,16^ and mutational and DNA methylation class results were obtained from the medical record. MGMT promoter status was obtained from the clinical records and was assessed as part of the clinical care by clinically validated pyrosequencing (Qiagen). The study was approved by the NYU Institutional Review Board.

### Data Assembly

Patient clinical and laboratory records classified as IDH-wild type glioblastoma based on NGS and CNS Tumor DNA methylation classifier were identified.^13^ Records were excluded due to either insufficient clinical follow-up or insufficient molecular results. Only patients who received standard of care chemoradiation per Stupp protocol were included in the analysis cohort. Cohort selection is shown in Supplement 1.

Average glucose was calculated using a time-weighted approach using all plasma glucose measurements between tissue diagnosis and date of death (or last follow-up, at which survival is censored). Each random glucose measurement was weighted by the length of time between it and the next record or censor date and normalized by the total length of follow-up. A similar approach was used to calculate average daily dexamethasone dosage. Steroid dosing documented in the medical record were verified against inpatient medication administrations and prescription records as available. Patients were grouped into tertiles based on the time-weighted average glucose values. When comparing progression free survival, average glucose and daily dexamethasone dose were calculated based only on values between diagnosis and progression. Additional clinical variables acquired included age, sex, Karnofsky performance score (KPS), prior diabetes, and prior cardiovascular risk factors defined as a history of hypertension (HTN), hyperlipidemia (HLD), coronary artery disease (CAD), or cerebrovascular accident (CVA).

### Survival Analysis

Continuous and categorical baseline attributes were compared using Wilcox rank sum and Chi-square tests, respectively. Univariate survival differences were compared using log-rank tests. Differences in overall and progression free survival were further interrogated using multivariate cox proportional hazard analyses. All *P*-values reflect two-sided tests with significance defined at *P* < .05. Statistical analyses and visualizations were performed using R software package (version 3.6.1) and GraphPad Prism 8.

### DNA Methylation Analysis

DNA methylation profiling was performed on all cases using Illumina EPIC array or 450K array. Raw IDATs generated from iScan were processed and analyzed using Bioconductor R package *Minfi*.^17^ Since samples were profiled on two different arrays, the data was combined to analyze overlapping probes that were present on both the arrays. Samples were checked for their quality using mean detection *P*-values (det *P* < .05). The probes were normalized using quantile normalization and corrected for background signal. Sex probes and SNPs were removed as part of normalization. Batch effects were corrected across the samples using *ComBat* function from *sva* R package.^18^ Beta values were obtained and differential methylation analysis was performed across all the three subclasses of GBM (RTK I, RTK II, Mesenchymal) using *dmpFinder* function from Minfi package. Probes with FDR cutoff (*q* < 0.05) were considered as most significantly variable probes. Beta value < 0.2 means Hypomethylation and Beta value > 0.8 means Hypermethylation.

The 10 000 most significant variable probes were used to generate a supervised heatmap using *ComplexHeatmap* package.^19^ Euclidean distance method was used for generating the distance matrix since it is the best method for continuous data and ward. D2 method was used for hierarchical clustering since it minimizes the within cluster variances resulting in compact clusters. Both these methods were used together to generate supervised heatmap. To have a clear visualization of these cluster subgroups in the heatmap, K-means of three clusters was applied to identify each subgroup clearly on the heatmap.

### Functional Pathway Enrichment Analysis

To find the most enriched signaling pathways in RTK I and Mesenchymal groups, the most significant differentially methylated probes (top 10 000) between High vs Low glucose patients were passed through the *ClusterProfiler* R package for KEGG enrichment.^20^ The dot plots represent ratio of genes (*x*-axis) involved in each signaling pathway (*y*-axis) of KEGG database.^21^ Size of the dots corresponds to gene counts and the color denotes the significance level.

### Metabolite Profiling

Approximately 10–20 mg of each sample were collected and placed in individual micro-centrifuge tubes on dry ice. For each sample, 600 µL of LC/MS grade methanol containing internal standards was added, followed by 300 µL of LC/MS grade water, and lastly 400 µL of chloroform. Bead-based homogenization was then performed using the Bertin Precellys 24 homogenizer. The samples were microcentrifuged at max speed for 10 min at 4°C, and the two subsequent layers were collected separately and SpeedVac dried. Metabolite samples were stored at −80°C prior to analysis.

Data preprocessing, batch correction and differential metabolomic analysis were performed using *MetaboAnalyst (version 3.2.0)* R package.^22^ Median normalization and log-transformation was applied to the processed peak intensities across the samples. This normalized data was then subjected to batch correction to remove any unwanted batch effects in the cohort. *EigenMS* batch effect correction method was applied to remove the batch effects since it aims at preserving the original differences between groups while removing the bias from the data.^23^ Missing values were imputed using limit of detection (LOD) values. Unsupervised PCA was generated using the normalized batch corrected data to look for any variances. To identify differences between binary low or high glucose samples, differential metabolomic analysis was carried out. Volcano plot was generated using the differentially significant metabolites (*P* value < .0.5). To generate the heatmap, *Z*-score was calculated using the normalized batch corrected data and the plots were generated using *ComplexHeatmap* R package.^19^

## Results

### Cohort Characteristics

A total of 89 patients with IDH-wildtype GBM diagnosed between 2014 and 2020 met the inclusion criteria (Supplement 1). Median age of patients in the cohort was 64 (range 29–86, Table 1). The cohort had slightly more patients with tumors that were MGMT unmethylated and EGFR-amplified (64% and 59%, respectively). While tumors were distributed among various methylation classifier subclasses, RTK II was the most common tumor subclass (36%). Median overall survival (OS) and progression free survival (PFS) were 18.0 and 17.6 months, respectively. No patients were diagnosed with new-onset diabetes or started on long-term anti-hyperglycemic treatments during follow-up. All patients succumbed to progressive disease except two. One patient passed away from a subdural hemorrhage after a traumatic fall and a second patient passed away after developing a massive pulmonary embolism.

**Table 1.** Clinical cohort characteristics

	Median (range) or %
Age	64 (29–86)
Sex (% male)	67.4%
MGMT methylated	36%
EGFR-amplified	59%
RTK I	29.2%
RTK II	36%
RTK III	1.1%
Mesenchymal	29.2%
Midline	4.5%
Gross or near total resection	49%
Median OS (mo)	18.0
Median PFS (mo)	7.6

For survival analyses, patients were divided into three equal groups for comparison based on average time-weighted glucose derived using random plasma glucose values between date of pathology and last follow-up: tertile one (<100 mg/dL), tertile two (100–115 mg/dL), and tertile three [>115 mg/dL (Supplement 2)]. Frequency of glucose measurements were similar across glucose tertiles, with averages of 11.7–13.0 days between each random plasma glucose value.

Comparison of clinical characteristics across glucose level tertiles revealed no significant differences with regards to KPS, average time-weighted dexamethasone dose, EGFR amplification, MGMT methylation, or methylation classifier subclass distribution (Table 2). Average age was higher in tertile three (*P* = .008, Table 2). While the number of patients with a pre-GBM diagnosis of diabetes was slightly higher in tertile three, these patients represent a small portion of the clinical cohort with only 7 out of 89 patients (7.9%).

**Table 2.** Cohort characteristics by glucose groups

	Average time-weighted glucose			
	<100 mg/dL	100–115 mg/dL	>115 mg/dL	*P*-val
*n*	30	29	30	
Age (per year)	60 (50–66)	58 (49–71)	69 (61–75)	**0.008**
Time-weighted glucose (before 1st progression)	94 (87–97)	106 (101–112)	127 (119–141)	**1e−12**
KPS	90 (80–90)	90 (80–90)	80 (70–90)	0.35
Time-weighted dexamethasone dose (mg/day)	1.3 (0.6–1.5)	1.3 (0.4–1.4)	2.5 (1.5–4.7)	0.12
EGFR amplification by FISH	14	17	16	0.65
MGMT methylated	12	10	10	0.55
Subclass				
RTK I	8	8	10	0.83
RTK II	14	11	7	0.16
RTK III	0	0	1	0.37
Mesenchymal	7	9	10	0.67
Midline	1	1	2	0.78
Median OS (mo)	21.2	17.1	14.1	**0.015**
Median PFS (mo)	7.0	7.1	9.3	0.41
Average number of days between glucose measurements	13.0 (9.8–22.5)	12.8 (11.3–21.5)	11.7 (7.0–18.6)	0.24
Pre-diagnosis cardiovascular risk factors (HTN, HLD, CAD, CVA)	12	20	21	**0.03**
Pre-diagnosis diabetes	0	2	5	0.05

P-values less than 0.05 are bolded.

### Impact of Hyperglycemia on Survival is Associated With Tumor Subclass

**Figure 1.**
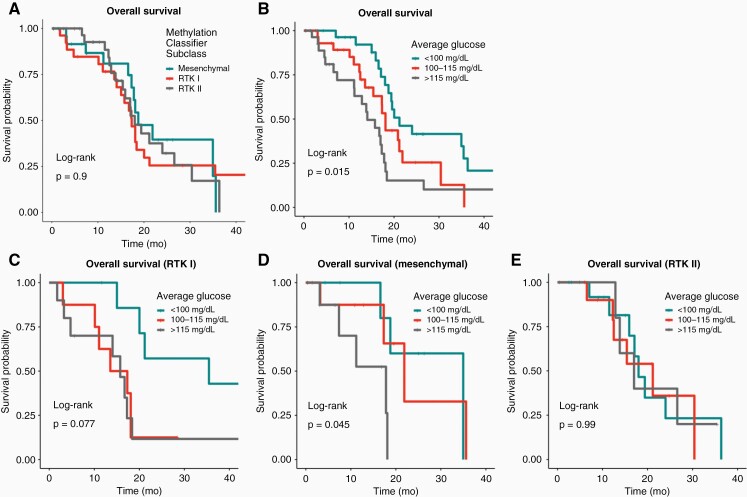
Overall survival in GBM patients. (A) OS is not affected by methylation classifier subclass. (B) OS decreases with higher average glucose values (21.2 to 17.1 to 14.1 months). (C, D) Glucose affects survival in RTK I and Mesenchymal tumors, but not RTK II tumors (E).

Overall survival (OS) decreased with higher average glucose values, with median OS decreasing from 21.2 to 14.1 months in tertile one to tertile three (log-rank *P* = .015, Figure 1B). In contrast, OS was not different between different methylation subclasses (log-rank *P* = .9, Figure 1A). In contrast, when analyzing serum glucose in specific molecular subtypes, higher glucose tertiles were showed strong trend towards worse OS in RTK I (log-rank *P* = .08, Figure 1C) and mesenchymal subclass (log-rank *P* = .05, Figure 1D), but not RTK II (log-rank *P* = .99, Figure 1E). From tertile one to three, median OS decreased from 35.5 to 15.8 months in RTK I tumors and decreased from 35.0 to 17.8 months in Mesenchymal tumors. Our cohort did not contain sufficient numbers of GBM RTK III and Midline subtype GBMs for analysis of the survival.

To further elucidate the role of blood glucose on survival, a multivariate cox model was employed to adjust for the effects of age, KPS, dexamethasone dose, and MGMT status. Higher glucose remained significantly associated with poorer survival (adjusted hazard ratio [aHR] = 5.2, *P* = .02, Table 3). Higher age, lower KPS, higher average dexamethasone dose, MGMT non-methylation, and methylation classifier subclass were also significantly associated with survival. The interaction of methylation subclass with glucose was significant (analysis of deviance, *P* = .03).

**Table 3.** Multivariate regression identifies significant associations between OS and average blood glucose after adjusting for confounders

	aHR	95% CI	*P*-val
Age (per year)	1.07	1.03–1.10	8.5e−5
KPS	0.95	0.93–0.98	4.8e−4
Average dexamethasone (per mg/day)	1.57	1.33–1.85	1.2e−7
MGMT unmethylated	4.55	2.03–10.1	2.2e−3
Average glucose			
<100 mg/dL	1	Ref	–
100–115 mg/dL	16.8	4.15–67.9	7.6e−5
>115 mg/dL	5.2	1.34–20.5	0.02
Methylation classifier subclass			
RTK I	1	Ref	–
RTK II	12.4	3.17–48.3	3.0e−4
Mesenchymal	10.9	2.06–57.2	4.9e−3
Other	11.6	1.14–118.2	0.04
Subclass * glucose interaction			
RTK II * 100–115 mg/dL	0.06	0.009–0.39	0.003
RTK II * >115 mg/dL	0.19	0.02–0.84	0.033
Mesenchymal * 100–115 mg/dL	0.07	0.008–0.57	0.01
Mesenchymal * >115 mg/dL	0.44	0.06–3.28	0.43
Other * 100–115 mg/dL	0.13	0.005–3.0	0.20
Other * >115 mg/dL	1.17	0.07–18.9	0.91

### No Discernible Epigenetic or Metabolomic Differences Between GBMs in Patients With Different gGlucose Levels

**Figure 2.**
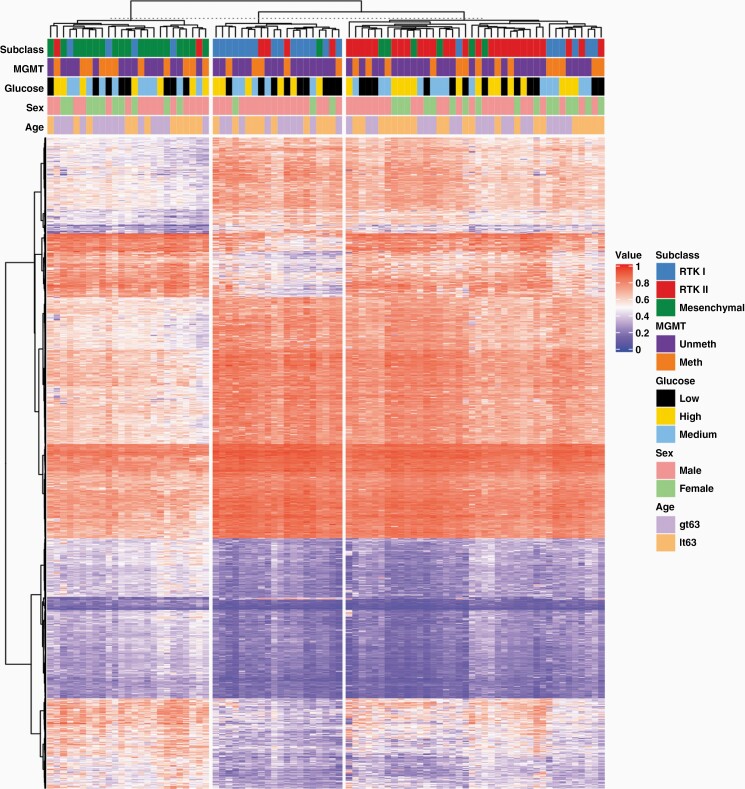
DNA methylation clustering shows subgroups based on methylation subclass but not glucose groups. Tumors arising in high circulating glucose environments do not form a unique DNA methylation subgroup. Methylation clusters exhibit heterogeneous distribution of MGMT methylation, sex, and patient age. lt63, age less than 63; gt63, age greater than 63.

To further understand the seemingly differential role of glucose across tumors of different methylation subclasses, methylation phenotype of 84 tumors classified as either mesenchymal, RTK I, or RTK II were further interrogated for tumor differences. Glucose groups were simplified into high vs low binary divisions to reduce comparator groups. Semi-supervised clustering by methylation classifier subclass showed no stratification by glucose levels groups, MGMT methylation, sex, or age (Figure 2).

**Figure 3.**
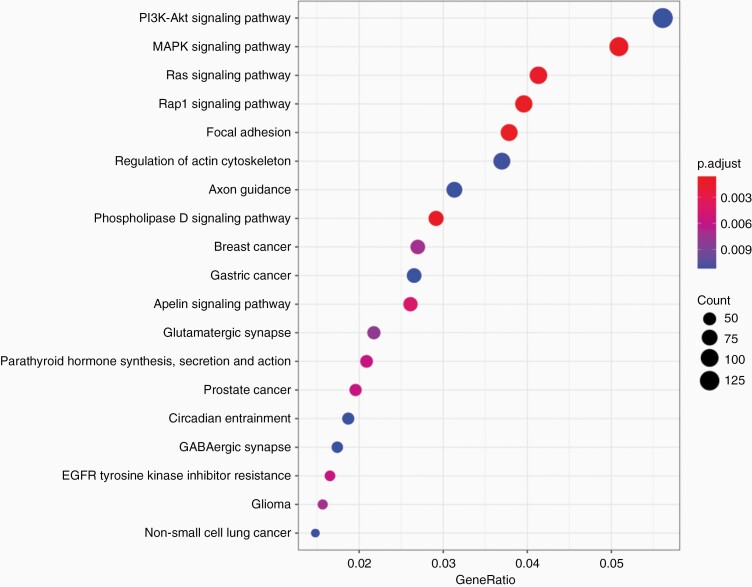
Pathway analysis of RTK I and Mesenchymal tumors using DNA methylation. Differential methylation comparing high vs low glucose environments among tumors identifies enrichment of PI3K, MAPK, and Ras signaling pathways in high glucose tumors.

Because of lack of DNA methylation clustering by glucose levels, we next performed a supervised analysis of methylation subclasses that exhibited differential OS by glucose levels to elucidate the signaling differences between GBMs in high and low systemic levels of glucose. Pathways analysis using differentially methylated probes (DMPs) comparing high glucose RTKI and Mesenchymal vs low RTKI and Mesenchymal GBM demonstrated enrichment in PI3K-AKT signaling, MAPK signaling, among others (Figure 3). Enrichment in MAPK signaling was also seen when comparing top variably methylated probes between high and low glucose among Mesenchymal and RTK I tumors separately (Supplement 3) suggesting that MAPK pathway is upregulated in tumors growing in high glucose macroenvironment.

To elucidate whether subgroup-specific differences in the role of glucose may be related to intratumoral metabolomics, bulk tumor levels were examined for 23 samples. Across 133 available metabolites, there were no metabolites that were significant based on FDR values (Supplement 4).

## Discussion

Using institutional retrospective data with long-term follow-up and detailed analysis of routine clinical laboratory glucose data, we found that that hyperglycemia was an independent factor portending poor prognoses in GBM. In a retrospective cohort treated with Stupp standard of care, higher blood glucose was associated with worse survival even after controlling for known significant clinical characteristics including: MGMT promoter methylation, clinical performance status (KPS), steroid use, and age. Furthermore, the role of hyperglycemia was different across three main molecular subtypes of GBM defined by methylation classifier. Higher average glucose values were associated with worse OS in RTK I and Mesenchymal IDH-wt GBM, but not in RTK II GBM. While HgbA1C levels, often interpreted as a rolling average of serum glucose, are not routinely measured in GBM patients, future studies on the role of serum glucose in GBM patients may incorporate HgbA1C measurements.

While there were no discernible differences between the overall methylation landscape of tumors in different glucose environments, a supervised pathway analysis identified PI3K-AKT, MAPK, RAS signaling pathways in high glucose tumors. Interestingly there were no differences in tumor metabolite levels that may explain our observation, although that may be due to overall low number of tumors for metabolic analysis. However, the lack of intrinsic differences among GBMs in high and low level glucose environments suggests a systemic lowering of glucose to improve survival.

There are numerous mechanisms by which glucose may affect patient survival. One series of explanations are centered on the direct role of glucose on tumor proliferation via increased substrate for glycolytic metabolism. Glioblastoma, similar to many other solid tumors, are able to exploit glucose-dependent metabolism even in the presence of oxygen to promote cell proliferation.^24^ As intracerebral glucose concentrations increase with rising plasma glucose, a finding that is exaggerated in patients without prior history of hyperglycemia, tumors in patients with high systemic blood glucose may have greater uptake of metabolic substrates and thus proliferation.^25–27^ A second possibility is that hyperglycemia influences survival in GBM through increased insulin levels. GBM cells expresses the same insulin receptors to those seen in peripheral tissues, therefore hyperglycemia-induced hyperinsulinemia may increase insulin signaling in cancer cells to have an effect that is independent of tumor metabolism.^28^ Hyperinsulinemia itself has been shown to also facilitate tumor growth via stimulation of the insulin-like growth factor cascade.^29,30^ This would be consistent with the upregulation of PI3K-AKT and MAPK signaling pathways observed, which would also be enhanced by insulin signaling.^31^ This possibility would also be consistent with the lack of correlation between glucose level and survival in the RTK II subclass, where EGFR amplification would serve a similar function to increased insulin signaling. While we were unable to elucidate variations in tumor metabolite levels, limitations of our approach include the lack of tissue after initial treatment, the use of bulk tumors without an infiltrating edge, and low number of tumors. It is possible that during the course of disease, more differences in tumor metabolite levels emerge as the tumor interacts with the surrounding glucose environments.

The adverse effects of hyperglycemia on patient survival may have non-tumor dependent effects. Patients with more aggressive tumors may require higher doses of steroids to alleviate symptoms of cerebral edema and thus develop steroid-induced hyperglycemia, especially in the palliative therapy scenario.^32^ However, our analysis demonstrates that average glucose is an independent risk factor for poor survival even after controlling for the average time-weighted steroid dose.^33,34^ Hyperglycemia or a history of diabetes may also increase the risk of other complications, such as infections, that affect mortality.^35,36^ However, only a small minority (7.9%) patients had a pre-GBM diagnosis of diabetes. The vast majority of patients had average blood glucose levels within the range of what is considered normal.^37^ The two patients in our cohort that succumbed to reasons not directly related to progressive intracranial disease passed away from a traumatic fall and pulmonary embolism, which are not highly associated with hyperglycemia. Therefore, it is unlikely that the increased mortality associated with hyperglycemia is related to non-tumor effects.

While we were unable to explain differences in tumor subclasses that relate to glucose outcomes, our results suggest several possibilities. The lack of overall methylation phenotype differences between tumors in different glucose environments suggest a differences in how different subclasses utilize glucose utility rather than intrinsic features specific to a subclass phenotype. When focusing on tumors that did exhibit differential responses to glucose environments, we noted an enrichment of high-level signaling pathways such as MAP-Kinase, PI3K, and Ras signaling, suggesting increased activity of these pathways in tumors within high glucose environments. RTK II tumors demonstrate greater enrichment for EGFR amplification,^12^ which, in other cancers, has been shown to maintain intracellular glucose levels through interaction with sodium glucose transporters.^38,39^ A similar feature may be seen in EGFR-amplified GBM tumors which may preferentially allow RTK II tumors to overcome relatively low ambient glucose levels by actively pumping the substrate into cells. In comparison, other subclasses may predominantly rely on passive glucose transporters, rendering these cells more dependent on the concentration of glucose available.

The differential role of hyperglycemia among different tumor subclasses may explain why prior studies on lowering blood glucose have yielded mixed effects on survival. Given that RTK II tumors, which are most similar to “Classical tumors” in TCGA datasets, do not exhibit differential survival based on glucose environments, and are the most common molecular subtype, studies with high proportions of RTK II tumors may be unable to detect the differences in survival by glucose.^40^ Our data further highlights the heterogeneity of GBM and the need to interpret clinical findings in the context of tumor specific molecular attributes. As GBM treatment becomes increasingly personalized and guided by molecular characteristics, rather than excluding RTK II tumors from trials investigating the effect of lowering glucose, DNA methylation sub-classification may be incorporated as an additional stratification variable to better understand if and how glucose-lowering benefits patients. While prior studies have not been able to demonstrate a prognostic role of tumor subclass, the differential role of glucose in various methylation classifier-determined subclasses may offer insight into a patient’s disease course. Given the relative ease and accessibility of DNA methylation profiling over other methods, wide-spread adoption of methylation profiling represents a more feasible option for personalized treatment decision-making.

## Conclusion

Higher average glucose values were associated with worse OS in RTK I and Mesenchymal IDH-wildtype GBM, but not RTK II. There were no discernible epigenetic or metabolomic differences between tumors in different glucose environments, suggesting a potential survival benefit with systemic glucose-lowering in selected molecular subtypes. The high proportion of RTK II tumors in GBM cohorts may conceal the detrimental effect of high glucose or benefit of lowering glucose and GBM clinical trials should incorporate molecular stratification by reproducible and widely adopted method such as DNA methylation.

## Supplementary Material

vdac163_suppl_Supplementary_Material
