# Supplementary material for: Association of hyperglycemia and molecular subclass on survival in IDH-wildtype glioblastoma
Source: Neurooncol Adv. 2022 Oct 11;4(1):vdac163. doi: 10.1093/noajnl/vdac163 (PMC9653172; doi:10.1093/noajnl/vdac163)

Supplement 1. Cohort selection.





Supplement 2. Average time-weighted glucose distribution. (A) Distribution of average time-weighted glucose separated into tertiles. (B-D) Representative glucose trajectories from diagnosis to last follow-up for different glucose tertiles.


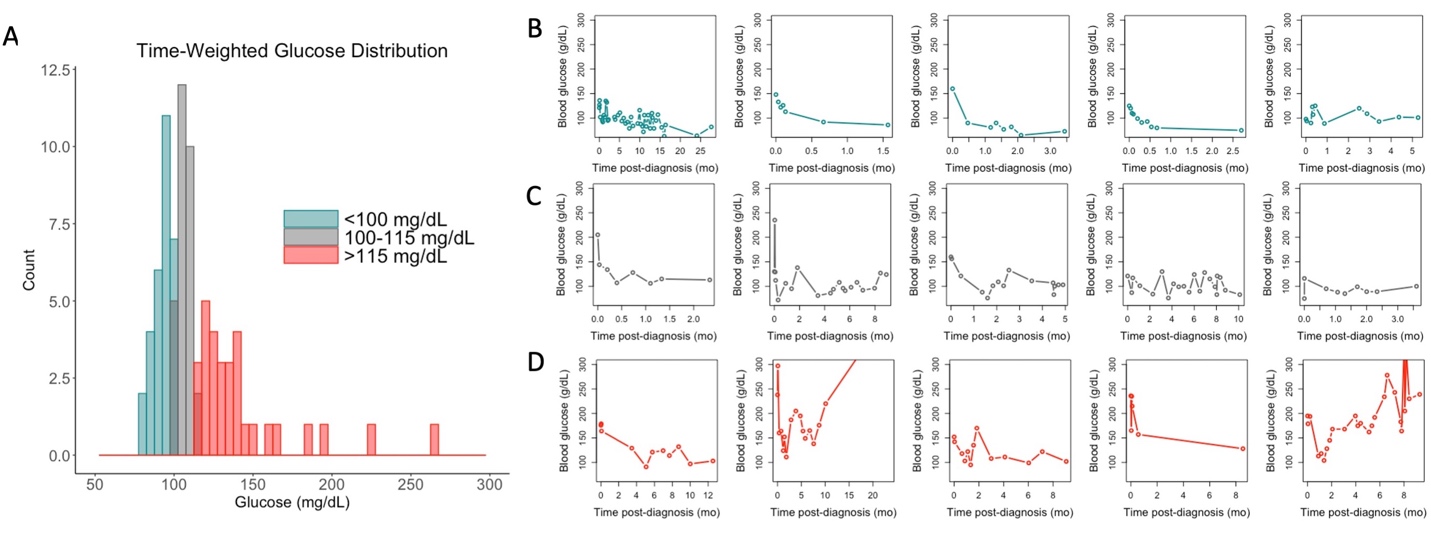


Supplement 3. KEGG for (A) Mesenchymal and (B) RTK I tumors.


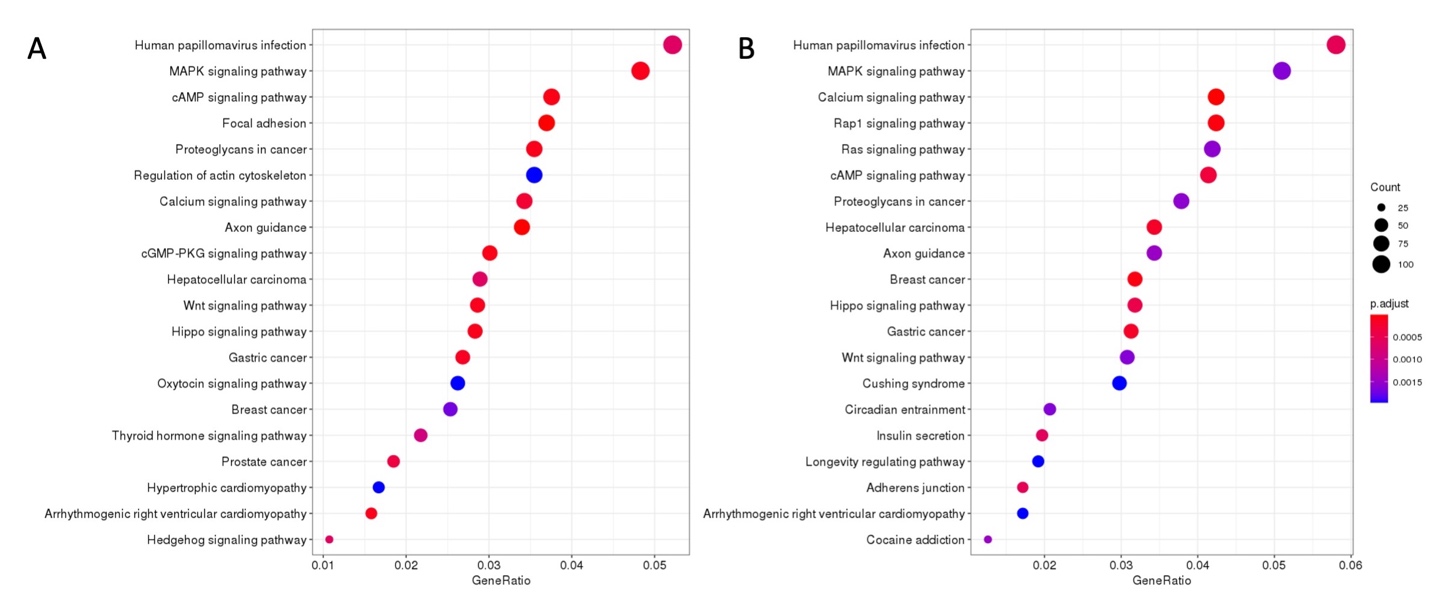


Supplement 4. Bulk tumor metabolite levels.


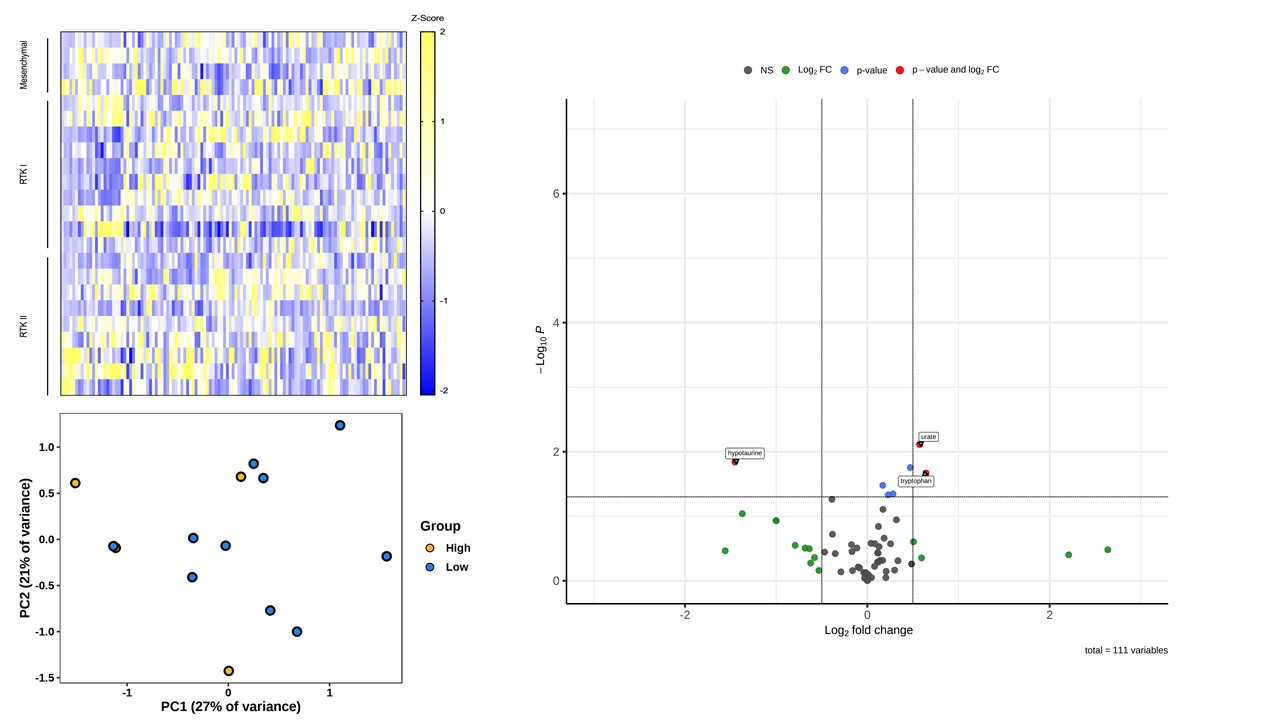

Supplement: vdac163_suppl_Supplementary_Material [file vdac163_suppl_supplementary_material.docx]
